# Soft Sensor with Deep Learning for Functional Region Detection in Urban Environments

**DOI:** 10.3390/s20123348

**Published:** 2020-06-12

**Authors:** Yicao Ma, Shifeng Liu, Gang Xue, Daqing Gong

**Affiliations:** School of Economics and Management, Beijing Jiaotong University, Beijing 100044, China; 19120617@bjtu.edu.cn (Y.M.); shfliu@bjtu.edu.cn (S.L.); 19113032@bjtu.edu.cn (G.X.)

**Keywords:** functional region, POI (point of interest), smart card, deep learning, soft sensors

## Abstract

The rapid development of urbanization has increased traffic pressure and made the identification of urban functional regions a popular research topic. Some studies have used point of interest (POI) data and smart card data (SCD) to conduct subway station classifications; however, the unity of both the model and the dataset limits the prediction results. This paper not only uses SCD and POI data, but also adds Online to Offline (OTO) e-commerce platform data, an application that provides customers with information about different businesses, like the location, the score, the comments, and so on. In this paper, these data are combined to and used to analyze each subway station, considering the diversity of data, and obtain a passenger flow feature map of different stations, the number of different types of POIs within 800 m, and the situation of surrounding OTO stores. This paper proposes a two-stage framework, to identify the functional region of subway stations. In the passenger flow stage, the SCD feature is extracted and converted to a feature map, and a ResNet model is used to get the output of stage 1. In the built environment stage, the POI and OTO features are extracted, and a deep neural network with stacked autoencoders (SAE–DNN) model is used to get the output of stage 2. Finally, the outputs of the two stages are connected and a SoftMax function is used to make the final identification of functional region. We performed experimental testing, and our experimental results show that the framework exhibits good performance and has a certain reference value in the planning of subway stations and their surroundings, contributing to the construction of smart cities.

## 1. Introduction

The increasing population in urban areas has led residents to demand more for daily life and travel. To meet the needs of residents and build better smart cities, governments need to use the Internet of Things and communication technologies to obtain real-time data for further decision-making and planning [1,2]. One of the most important problems is identifying urban functional regions. Urban functional regions were first proposed in the Athens Charter, which claims that planners should address four types of city areas: the residential region, the work region, the recreation region, and the transportation region. With the development of cities around the world, other functional regions have emerged that make the urban spatial structure more complicated, and these new functional regions vary with the specific features of each city [3,4]. Urban functional regions can be defined by some types of activities or spatial interactions that may occur in a region [3]. In urban areas, one of the fastest growing means of transportation are subways. The rapid development of the subway has provided residents with great convenience. Due to the diversity of individual purposes and preferences, different subway stations have gradually reflected their unique functions [5,6], and the same station functional groups show similar rules [7]. The accuracy of the classification results for subway stations is closely related to the effect of this approach, and accurate classification shows advantages in strengthening the use of stations and surrounding land resources. Good results of the approach applied can relieve traffic pressure and meet the needs of residents so that the government can better formulate relevant development policies.

Previous studies on the classification of functional areas for subway stations are rare, while there are more studies on the division of urban functional areas. Methods based on geographic information systems (GISs) have been proposed for the identification of urban functional areas [7]. Furthermore, remote sensing (RS) and RS images with high spatial resolution (HSR) have been widely used to study land use and functional regions [8,9,10]. Geodemographic classification methods have also been used to identify functional areas based on social demographic data in certain areas [11]. Later, various types of geospatial big data, such as points of interest (POI), vehicle trajectories, mobile phone signals (MPSs), and social media have been used for research [12,13,14]. Among them, POI data are widely used because these data provide a large amount of building information, and have been widely used in urban boundary planning and urban spatial structure research [15,16,17]. However, more precise land-using map requires more data, and so Lan et al., Long et al., Hong et al. and Gan et al. have started to combine POI data and smart card data (SCD) in their research; SCD contains considerable amounts of passenger-related information, and it has increasingly been used in research [18,19,20,21]. Nonetheless, more than two kinds of data can contribute to a more comprehensive and authoritative classification result, making greater contributions to decision making and society. SCD and POI data are objective data, and previous research has lacked subjective data, such as people’s scores on surrounding businesses. Subjective data include emotions, so they can truly reflect people’s attitudes and feelings. The ultimate purpose of the research is to benefit society and residents; therefore, it is important to consider subjective data related to residents to better achieve research goals. To solve this problem, this paper collects Online to Offline (OTO) e-commerce platform data in the analysis. OTO e-commerce is a platform that contains information about various businesses. People can see the basic introductions related to this business, and they can rate on OTO platform as well as fill out a public evaluation based on their service experience.

In this paper, to promote identification accuracy, we use three kinds of data. SCD records show the overall passenger rules of the stations, POI gives the static geographic information, and OTO indirectly reflects people’s evaluation of the stations. The contributions of this paper are as follows. First, to reduce the impact of a lack of data diversity on the results, this paper uses more kinds of data by collecting data from the OTO platform, in addition to SCD and POI data. Expanding the breadth of the data and the research dimensions can lead to more rigorous results. Second, this paper constructs a passenger flow feature map that contains specific information of each subway station and can reflect their characteristics. Third, this paper describes the creation of a hybrid neural network approach that allows input vectors and maps to simultaneously consider the features of data and the need for data. The two-stage framework includes the passenger flow stage and the built environment stage. In the first stage, SCD features are transferred into a feature map and then this map is fed into an identification model. Then the residual neural network (ResNet) model is used. In the second stage, POI and OTO data features are extracted and processed in a deep neural network with stacked autoencoders (SAE–DNN) model. Finally, the output of two stages is combined and a SoftMax function is used to make final identification.

The rest of this paper is organized as follows. In Section 2, related work is discussed. This section states the achievements and deficiencies of previous research. Section 3 introduces our novel method in detail and how we process different data. In Section 4 we introduce our study area and the data we collected and compare different methods, hyperparameters, and datasets, and present the experimental results. In Section 5, we summarize the content of the paper and draw a conclusion.

## 2. Related Work

### 2.1. Image Recognition

Remote sensing images are widely used to help identify land use and urban functional regions [22,23]. Remote sensing techniques are more effective in identifying the physical features of the land, such as land surface features, but they do not have many abilities to identify human social activities [24,25,26,27]. The division of urban functional areas must consider actual human activities more, which will change over time and cannot be fixed [28], instead of only relying on the land surface features or the plans proposed by the government. Hu et al. [29] noted that urban areas of interest (AOIs) are related to people in the urban environment. These areas are closely related to humans, and people often visit these places. For example, social media data, such as pictures, can record human connections to these environments. These AOIs can be defined by human activities, providing useful information for urban planners and decision makers. However, this method also has limitations, such as failure to record all types of regions. Although current studies can identify more regions by combining high-level semantic information, it is still difficult to identify different human social activities [30,31].

### 2.2. Geodemographic Classification

Compared to remote sensing, geodemographics can be used to describe and analyze individuals by where they live, and geodemographic classification is a spatially explicit classification of socioeconomic data, contributing to describing the sociodemographic structure of urban regions [32,33]. Although there are still some problems with geodemographic classification, for example, a few dimensions cannot provide enough information to fully describe an area, this has still become a mainstream method [34]. Using this method can identify many functional areas, such as education, retail, medical, and work zones [35,36,37]. Martin et al. [38] used geographic areas to solve the problems derived from workplace population data. Many related studies have shown that this type of classification method is efficient, although there is still considerable opportunity for improvement [39].

### 2.3. Big Data and Smart Card Data

In more recent studies, POI data and SCD have been combined for research. Long et al. [40] converted the bus credit card data into two-dimensional time series data for each bus station’s flow and constructed a city functional area recognition model based on the bus credit card data and POI data. Liu et al. [41] proposed a method to automatically identify and characterize parcels using OpenStreetMap (OSM) and points of interest (POI) data. Wang et al. [42] presented a new model integrating geographic information systems (GIS) with artificial neural networks (ANNs) to predict multiple transitions among land use types and urban subclasses. Bao et al. [43] first used k-means clustering to divide bicycle sharing stations into five categories based on surrounding POIs and then combined them with SCD for Latent Dirichlet Allocation (LDA) analysis to infer the purpose of cycling. Wang et al. [44] proposed the semantic framework of IS2Fun by using doc2vec to derive the relationship between the travelers and the stations, to infer the function of the subway stations. Tang et al. [45] used SCD to derive passenger travel patterns and relied on the LDA model with POI data to separately obtain mobile semantics and location semantics. After standardization and other processes, the improved k-means algorithm was used to cluster and obtain the function of the subway stations. Zhao et al. [46] developed an identification of land-use characteristics using bicycle sharing data.

SCD includes too much information, but previous studies lack a methods such as a feature maps to integrate them together and display them clearly and easily. In addition, there are many studies that have combined more data or innovative new methods for exploration. For instance, Bao et al. [47] not only used bike-sharing trip data and POI data but also added bicycle infrastructure data, weather data, and sociodemographic characteristics. Some even combined land data with spatial information [48,49,50]. Zhai et al. [51] proposed the method of place2vec. However, in previous studies, POI data and SCD were basically used separately, and they were not input to a model together for calculation. Some research failed to achieve data diversity, and they rarely combined e-commerce platform data. For the analysis, POI data and SCD can help to divide the functional area, but the accuracy will be weakened.

Over time, a considerable amount of research has focused on land use in cities or the division of cities into functional areas. The methods used in this research have been changing. Image recognition was used mainly in the early stages to identify urban functional areas; however, it is difficult to finish complex identification tasks. Later, geodemographic classification became popular. This method combines more information, but it still has considerable potential to be improved. Recently, research has used big data and SCD, primarily due to comprehensiveness and timeliness. Nonetheless, the diversity of data needs to be considered more carefully so that accuracy can be increased.

## 3. Method

**Figure 1:**
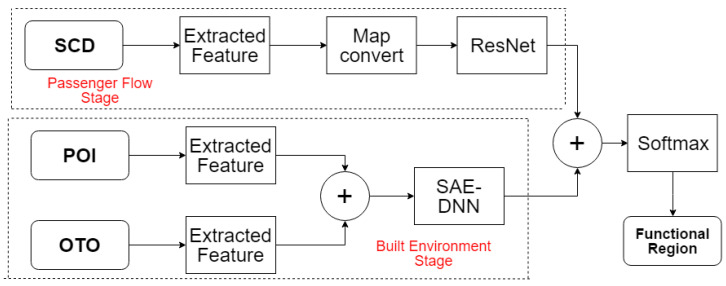
The overall framework.

As shown in Figure 1, we propose a two-stages framework, to identify the functional region of subway stations. In the passenger flow stage, the SCD feature is extracted and converted to a feature map, and a ResNet model is used to get the output of stage 1. In the built environment stage, the POI and OTO feature are extracted, and a deep neural network with stacked autoencoders (SAE–DNN) model is used to get the output of stage 2. Final we connect the outputs of two stages and use a SoftMax function to make the final identification of functional region.

### 3.1. Passenger Flow Stage

#### 3.1.1. SCD Processing

Several researchers have applied SCD to identify the functional region, but their methods chose some indicators of passengers and missed some high-level features of passengers. To obtain deep level features, this paper presents a method to create a passenger feature map which retains all the SCD records without indicators. To explore the rules of passenger flow at each station, taking a single station as an example, the SCD collected is counted by the hour, and the total number of people entering and leaving the station in each hour interval is recorded separately. Then, the changes in passenger flow at each station can be observed at different time periods. Weekdays and weekends are separated to count the number of people entering and leaving the station at different intervals on different days because the characteristics of passenger flow at some stations differ greatly on weekdays and weekends.

Assume *i* is the index of entering station, *j* is index of the exiting station and the values of *i* and *j* are 1 to 358 (Beijing Subway System has 358 stations). The *i-*th station weekends’ inbound feature matrix *A* (*i*) and the *i-*th station weekends’ inbound feature matrix *M* (*i*) are as follows:

(1) $$A\left(i\right)=\left(\begin{array}{c} a\left(i,1\right)\\ \begin{array}{c} \ldots \\ a\left(i,j\right)\\ \ldots \end{array}\\ a\left(i,358\right) \end{array}\right),$$

(2) $$M\left(i\right)=\left(\begin{array}{c} m\left(i,1\right)\\ \begin{array}{c} \ldots \\ m\left(i,j\right)\\ \ldots \end{array}\\ m\left(i,358\right) \end{array}\right),$$

where *a* (*i*, *j*) represents the vector of the total number of people coming in from the *i*-th subway station and leaving from the *j*-th subway station on weekdays. *a* (*i*, *j*) = (*C* (*i*, *j*, 0), *C* (*i*, *j*, 1), …, *C* (*i*, *j*, *k*)), where *C* (*i*, *j*, *k*) represents the total number of people who enter from the *i*-th subway station and exit from the *j*-th subway station in the *k*-th period on weekdays. Where *k* represents time intervals, with an interval of one hour, and the value of *k* ranges from 0 to 23.

*m*(*i*, *j*) is similar to *a* (*i*, *j*), but it represents the vector of the total number of people coming in from the *i*-th subway station and leaving from the *j*-th subway station at different time periods on weekends. *m* (*i*, *j*) = (*P* (*i*, *j*, 0), *P* (*i*, *j*, 1), …, *P* (*i*, *j*, *k*)), where *P* (*i*, *j*, *k*) represents the total number of people who enter from the *i*-th subway station and exit from the *j*-th subway station in the *k*-th period on weekends.

In addition to recording the number of people entering a specific station, it is also necessary to know the number of people leaving the station. The *i-*th station weekdays’ outbound feature matrix *B*(*i*) and the *i-*th station weekends’ outbound feature matrix *N* (*i*) are as follows:

(3) $$B\left(i\right)=\left(\begin{array}{c} b\left(i,1\right)\\ \begin{array}{c} \ldots \\ b\left(i,j\right)\\ \ldots \end{array}\\ b\left(i,358\right) \end{array}\right),$$

(4) $$N\left(i\right)=\left(\begin{array}{c} b\left(i,1\right)\\ \begin{array}{c} \ldots \\ b\left(i,j\right)\\ \ldots \end{array}\\ b\left(i,358\right) \end{array}\right),$$

where *b*(*i*, *j*) represents the vector of the total number of people coming in from the *j*-th subway station and leaving from the *i*-th subway station on weekdays. *b* (*i*, *j*) = (D (*i*, *j*, 0), D (*i*, *j*, 1), …, D (*i*, *j*, *k*)), where D (*i*, *j*, *k*) represents the total number of people who enter from the *j*-th subway station and exit from the *i-*th subway station in the *k*-th period on weekdays. And *n* (*i*, *j*) is similar to *b*(*i*, *j*), but it indicates the vector of the total number of people coming in from the *j*-th subway station and leaving from the *i*-th subway station at different time periods on weekends. *n*(*i*, *j*) = (*Q* (*i*, *j*, 0), *Q* (*i*, *j*, 1), …, *Q* (*i*, *j*, *k*)). where *Q*(*i*, *j*, *k*) represents the total number of people who enter from the *j*-th subway station and exit from the *i-*th subway station in the *k*-th period on weekends.

After the above steps, four matrixes are obtained: weekday station-entering statistics, weekday station-exiting statistics, weekend station-entering statistics, and weekend station-exiting statistics.

*R* (*i*) is the input. To train the features in a convolutional neural network (CNN)-based model, assuming that the feature map of site *i* is *R* (*i*), the composition of the feature map is shown below:

(5) $$R(i)=\left(\begin{array}{c} A\left(i\right)\\ \begin{array}{c} M\left(i\right)\\ B\left(i\right) \end{array}\\ N\left(i\right) \end{array}\right),$$

**Figure 2:**
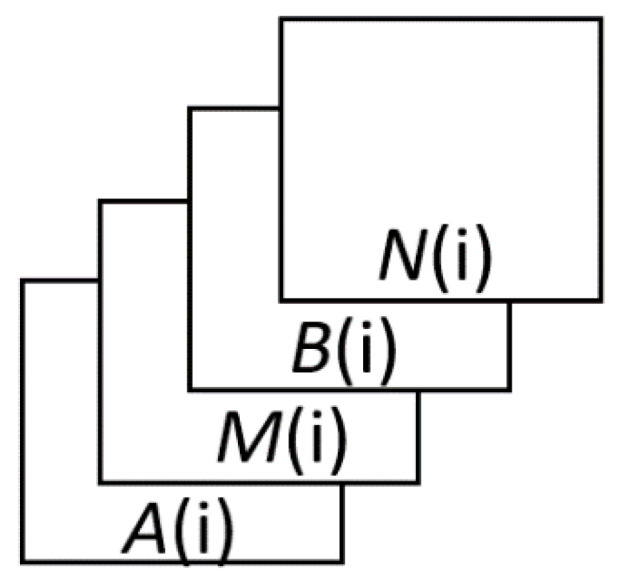
The form of *R*(*i*).

The passenger flow feature map *R*(*i*) is a superposition of four matrices, forming a 3D vector (Figure 2).

#### 3.1.2. ResNet Model

**Figure 3:**
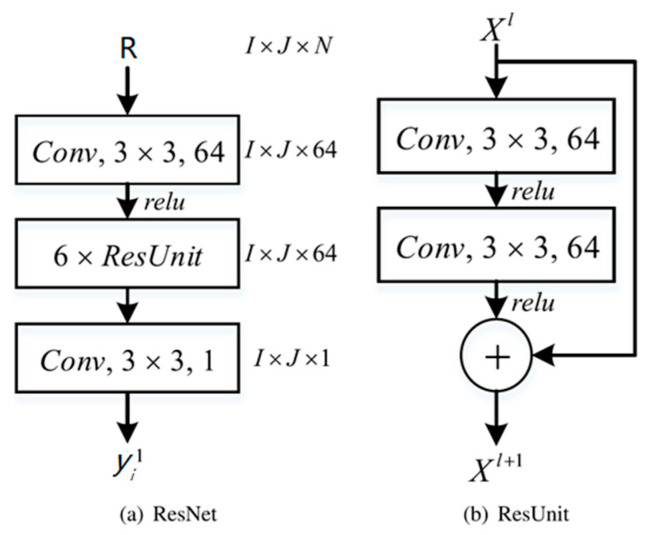
The structure of the prediction model.

To identify the travel pattern of passenger flow, *R*(*i*) is used as an input of the identification model, as:

(6) $$y_{i}^{1}=e\left(R\left(i\right)\right),$$

where *e* denotes to a machine learning identification model. In this section, due to the good learning ability of the residual neural network (ResNet) [52,53,54], it is used to construct the identification model. ResNet is a special CNN model which make use of very deep hidden layers by using residual units (ResUnits), as

(7) $$X^{l+1}=X^{l}+F\left(X^{l}\right),$$

where $X^{l}$ is the input of the *l*-th ResUnit, $X^{l+1}$ denotes to the output of the *l-*th ResUnit and F(·) represents to the residual function. As shown in Figure 3, 14 layers are made up of 6 ResUnits, an input convolution layer, and an output convolution layer to construct the prediction model. In each ResUnit, we use 64 filters of 3 × 3 with zero-padding to stack two convolution layers. 64 filters are used in the input layer while one filter is utilized in the output layer. Consequently, we get an output as $y_{i}^{1}$.

### 3.2. Built Environment Stage

In the built environment stage, we introduce two kind of data, including POI and OTO; which could reflect the station feature in different dimensions. POI includes the static information of buildings, and OTO includes the dynamic information of passenger preference.

#### 3.2.1. Point of Interest Data Processing

The POI data are processed by using the latitude and longitude coordinates of both subway stations and all POI points in Beijing to calculate the number of POIs around different subway stations within a certain range and how many POIs each category has. Based on Cervero et al., Kuby et al., and Zhao et al. [55,56,57], for this study we chose to set the range as 800 m. Using the latitude and longitude distance formula, the distance between each POI point and subway station can be calculated. Taking each station as an example, only the businesses that are less than or equal to 800 m away from the station were considered, and the number was determined according to the category to which these businesses belong. Thus, the classification of different types of businesses around each station can be known. The *i-*th stations’ POI feature vector is defined as:

(8) $$Wi=(\begin{array}{ccc} S\left(i,1\right) & S\left(i,2\right) & \begin{array}{ccc} S\left(i,3\right) & \ldots & S\left(i,19\right) \end{array} \end{array}),$$

where S (*i*, *j*) represents the total number of *j*-th category of the *i*-th subway station.

#### 3.2.2. Online to Offline (OTO) Data Processing

OTO data can help in understanding the distribution of different types of businesses around subway stations and the overall satisfaction with these businesses. Taking a single subway station as an example, the process is as follows: count the number of businesses at different categories around this subway station first, then calculate an average of the ratings of the businesses in each category, and finally calculate a comprehensive average score for this subway station according to the weight of different categories. Assume *i* is a subway station, *m* is the category, *v*(*i*) = (*V* (*i*,1), *V* (*i*, 2), …, *V* (*i*, 8)), where *V* (*i*, *j*) represents the percentage of the m category of the *i*-th subway station.

*g*(*i*) = (*G* (*i*,1), *G*(*i*, 2), …,*G*(*i*, j)), where *G* (*i*, *j*) represents the average score of *j*-th categories of the *i*-th subway station, and *H* (*i*) represents the comprehensive score of the *i*-th subway station. The formulas are as follows:

(9) $$H\left(i\right)=\overset{8}{\underset{j=1}{\sum }}V\left(i,j\right)\times G\left(i,j\right),$$

The vector matrix *Z* of the final OTO data is of the form:

(10) $$Z_{i}\left(\begin{array}{ccc} V_{i} & G_{i} & H_{i} \end{array}\right),\;\;\;\;\;\;\;\;\;\;\;\;\;\;\;\;\;\;\;\;\;\;\;\;\;\;\;\;\;$$

#### 3.2.3. SAE-DNN Model

For suspected anomaly recognition, a deep neutral network with stacked autoencoders (SAE-DNN) model was built in this study. We conducted feature extraction about the feature vector in the SAE stage, and input the trained weight into the DNN, contributing to the high accuracy of suspected anomaly detection.

In unsupervised learning of efficient coding, an autoencoder, which is an artificial neural network, is used [58]. In order to solve the problem that ‘‘back propagation without a teacher”, Hinton and the PDP group became the first people to introduce the concept of the autoencoder [59]. Otherwise, the input data was used as the teacher [60]. Lately, autoencoders have played a pretty important role in learning generative models of data [61].

It is typically made up of three parts. The first part is an input layer, containing the input vector *X*, where $X=\{W_{i},Z_{i}\}$. The second part is a set of hidden layers, containing the transformed feature vector $H^{SAE}$, which is defined as an encoder shown in Equation (11). The final part is an output layer, including the reconstruction vector $R^{SAE}$, which is defined as a decoder shown in Equation (12).

δ and $\tilde{\delta }$ represent the linear and weighted combinations shown in Equations (12) and (13). The output vectors should match the input vectors, which have the same dimensions and values as the input vectors. *T*(·) denotes to the activation function. *Tanh* and *rectifier* are applied as the activation functions displayed in Equations (15) and (16), respectively [62].

(11) $$H^{SAE}=f\left(X\right)=T\left(\underset{i}{\sum }w_{i}x_{i}+b_{i}\right),$$

(12) $$R^{SAE}=g\left(X\right)=T\left(\underset{i}{\sum }\tilde{w_{i}}h_{i}+\tilde{b_{i}}\right),$$

(13) $$\delta =\underset{i}{\sum }w_{i}x_{i}+b_{i},$$

(14) $$\tilde{\delta }=\underset{i}{\sum }\tilde{w_{i}}x_{i}+\tilde{b_{i}},$$

(15) $$Tanh\left(\alpha \right)=\frac{e^{\alpha }-e^{-\alpha }}{e^{\alpha }+e^{-\alpha }},$$

(16) Rectifer(α)=max(0,α),

where input vector *X* represents a set of training datasets *{x*_1_, *x*_2_, *x*_3_, *…*,*x_n_}*; $H^{SAE}$ is a set of encoders *{h*_1_, *h*_2_, *h*_3_, *…*,*h_n_}*; *R* denotes a set of reconstruction results {r_1_, r_2_, r_3_, *…*,r_n_}; f(*X*) is the encoder function with weight *(w_i_)* and bias *(b_i_)*; g(*H*) represents the decoder function with weight ($\tilde{w_{i}}$) and bias ($b_{i}$); and α denotes δ or $\tilde{\delta }$.

The function of Equation (17) is minimizing the reconstruction error between the input vector *X* and the reconstruction vector $R^{SAE}$.

(17) $$L\left(X,R^{SAE}\right)=minL\left(X,RR^{SAE}\right),$$

The reason why we fine-tuned the parameters of $w_{i}$ and $b_{i}$, shown in Equations (18) and (19) to minimize the loss function *L*(*X*,$\;R^{SAE}$), is that we want to match the reconstruction results *R* and the input vector *X*.

(18) $$w_{i}{}^{\prime }=w_{i}-\eta \frac{\partial L\left(x_{i},r_{i}\right)}{\partial w_{i}},$$

(19) $$bi^{\prime }=bi-\eta \frac{\partial L\left(x_{i},r_{i}\right)}{\partial bi},$$

where $w_{i}$ and $w_{i}{}^{\prime }$ denote to the original weight and updated weight for the *i-*th node in each hidden layer; bi and $bi^{\prime }$ denote to the original bias and updated bias for the *i-*th node in each hidden layer; and η is the learning rate. Hence, we obtain an output as $y_{i}^{2}=R^{SAE}$.

### 3.3. Final Prediction

We connected the output of the above two stages, $y_{i}$ is the connection result,

(20) $$y_{i}=y_{i}^{1}+y_{i}^{2}$$

Then we used the SoftMax function to get the identification result, that is, the exponential normalization function, which is used in the multiclassification process. It maps the output of multiple neurons into the (0, 1) interval, which can be understood as a probability for multiple classifications.

### 3.4. Parameters Learning

The overall framework is to solve an optimization problem, the decision variables are the parameters of 2 stages, objective function is the mean squared error (MSE) of predicted value, as:

(21) $$\theta =arg\;min_{\theta }y_{i}-{\hat{y_{i}}}^{2},$$

where $y_{i}$ denotes the real value of the outbound flow of target station, and $\hat{y_{i}}$ denotes the predicted value of the outbound flow of target station. And θ denote to the parameters of 2 stages, which can be learned through the Adam optimizer via backpropagation.

## 4. Experiments

### 4.1. Study Area

**Figure 4:**
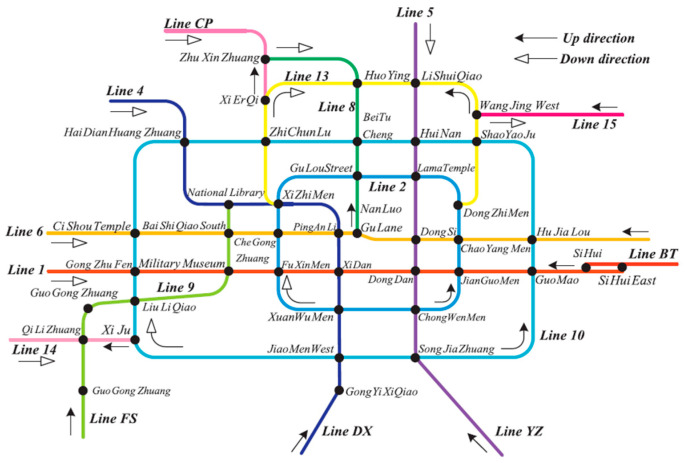
Beijing rail transit lines [49].

As the capital of China, Beijing is the national political and cultural center. It consists of 16 municipal districts and covers an area of 16,412 km^2^. As of 2016, the total population of Beijing reached 21.729 million. Beijing’s development prospects are becoming increasingly better, and numerous job opportunities and platforms constantly attract outsiders. As a result, Beijing’s resident population is also increasing. To better deal with the thriving development of Beijing and the rising demand for tourism, the local government officially opened and operated subway lines on 15 January, 1971, and Beijing became the first city in China to open a subway. An increasing number of subway lines have opened since, making it convenient for residents to travel and reducing traffic pressure. At the end of July 2017, there were 20 Beijing subway lines under construction, totaling 354.8 km^2^. It is expected that by 2020, the Beijing metro will form a rail network with nearly 30 lines and a total length of more than 1000 m. By the end of January 2020, the Beijing metro had a total of more than 380 stations serving the core areas, connecting the city center and suburbs (Figure 4).

### 4.2. Data Description

#### 4.2.1. Smart Card (SCD)

Smart card refers to the plastic card with embedded microchip which people swipe to get in and out of the subway station. Smart card data can provide information about the riding time and travel path of a passenger. The SCD record is derived from the Automatic Fare Collection System (AFC) of the Beijing metro and covers all the credit card data of the Beijing metro for up to six months. However, the specific private information of each card is not available.

This paper uses a full half-year SCD (from November 2017 to April 2018), approximately 8 million per day, excluding the problematic data of credit card records caused by subway equipment failures, such as an outbound station time earlier than entering station time. After filtering, these data serve as the data source for this paper.

#### 4.2.2. Points of Interest (POI)

The POI data for this research were downloaded from the Gaode Map (https://www.amap.com/) application programming interface (API). All 2019 POIs located in Beijing are collected, and all building information is divided into nineteen categories, including name, address, company, shopping, organization, infrastructure, construction and real estate, transportation, education, hotel, tourism service, food, car, life service, cultural venue, entertainment, medical care, banking and finance, sports and others. Each POI corresponds to a latitude and longitude coordinate, a large classification, and a small classification. Additionally, this paper also collects the latitude and longitude coordinates of all subway stations, for a total of 358.

#### 4.2.3. Online to Offline (OTO): Meituan

In addition to the subway SCD and POI data, this paper also uses crawler software to collect Meituan data as the OTO data. Meituan is a platform that contains information about various businesses. People can see the basic introductions related to this business, and they can rate on Meituan as well as fill out a public evaluation based on their service experience. On the Meituan page, different subway station names are entered, the crawler jumps to the page, and the first 31 pages of the business are selected, which is approximately 1000 records. The information contains the store names, ratings, and number of reviews of these businesses. To eliminate some duplicate business information, deduplication processing is carried out to obtain the final data. These stores have a total of 18 categories: online shopping, local shopping, home decoration, pet, wedding, cate, parenting, travel, training, entertainment, cinema, sports, life, health and beauty, food, hotel, real estate, and medical care. Some of them have similarities, so, after reorganization, they are divided into the following 8 categories: shopping, family, entertainment, life, food, hotel, real estate, and medical care.

#### 4.2.4. Ground Truth

This paper refers to Beijing’s Overall Urban Plan (2016–2035) issued by The People’s Government of Beijing Municipality [63]. Two kinds of classifications are used to test the model. jobs-housing based classification classifies all subway stations into four categories: residential, work, hybrid, and transportation. In addition, multi daily activities-based classification classifies all subway stations into eight categories: science education, health, leisure entertainment, sport, greenbelt, residential regions, work residential regions, and transport residential regions.

### 4.3. Experimental Setup

#### 4.3.1. Baseline Method

The framework we proposed is compared with a variety of competing methods grouped into the following categories:

The classical methods, liner regression (LR), random forest regression (RFR), and support vector regression (SVR). Deep learning methods, including deep neural networks (DNNs), and Conv based methods, convolutional neural networks (CNNs), ConvLSTM and ConvGRU.

#### 4.3.2. Evaluation Metrics

The mean square error (MSE) and *R*-square are chosen to represent the difference between the test value of subway station functional region and output value from the proposed framework.

(22) $$\mathrm{MSE}=\frac{1}{T}\sum_{i=1}^{T}{\left(\hat{y_{i}}-y_{i}\right)}^{2},$$

(23) $$R^{2}=1-\frac{\sum_{i=1}^{T}{\left(\hat{y_{i}}-y_{i}\right)}^{2}}{\sum_{i=1}^{T}{\left(\bar{y_{i}}-y_{i}\right)}^{2}}$$

where $y_{i}$ and $\hat{y_{i}}$ are the available ground truths and the corresponding predicted values, respectively; *T* is the number of all available ground truths, $\bar{y_{i}}$ denotes to the average value of $y_{i}$.

### 4.4. Result

#### 4.4.1. Comparison with Baseline Methods

We compare the overall performance between SAE-DNNs + ResNet and 19 baseline methods. As shown in Table 1, SAE-DNNs + ResNet achieves the lowest RMSE (40.25), and the highest $R^{2}$ (0.86) among all the methods, which is 18.82% (MSE), and 3.61% ($R^{2}$) relative improvement over the best performance among baseline methods. More specifically, methods including classical methods perform poorly, as they purely mine deep level features. SAE-DNN and ResNet further capture deep level features, and thus achieve better performance.

#### 4.4.2. Comparison with Variants of the Framework

**Figure 5:**
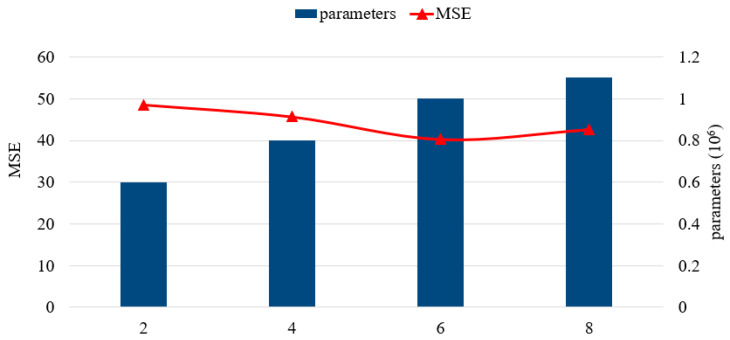
Number of residual units.

**Figure 6:**
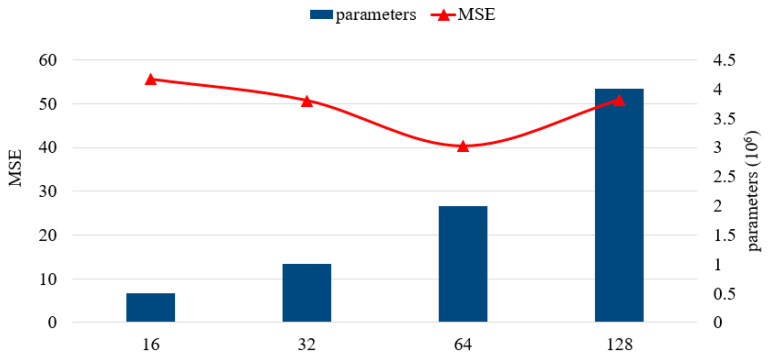
Number of filters.

We further analyzed the impact of hyperparameters in the framework, including the structure of SAE-DNNs and the residual units, and filters. In the following discussion, we change one hyperparameter while keeping other hyperparameters unchanged. Table 2 shows the performance of different SAE-DNNs structures, the structure of 4 hidden layers and [200, 400, 400, 200] hidden units perform best. As can be seen in Figure 5 and Figure 6, the RMSE first declines and then goes up with the increase of the quantity of residual units, or filters. We discovered that when six residual units and 64 filters are used, our method exhibited the best performance.

In this study, training data sets were divided into seven types to test whether multi-dimensional features can improve the performance of the prediction model. Table 3 illustrates that when using two kinds of data sources, the performances were better than only using one, and considering all three data sources, the model performed best. It proved that the functional region of subway station is not only determined by the passenger features, but only determined by the built environment features. Introducing both static information about buildings and dynamic information about passenger preference could promote the accuracy of the framework.

#### 4.4.3. Results Discussion

Table 4 shows a confusion matrix of jobs–housing based classification. The accuracy of work regions reached 0.876 and the accuracy of hybrid regions reached 0.902. However, the accuracy of residential regions and transport regions were lower than these, reaching 0.833 and 0.818 respectively. There are many shops and schools around some communities, leading to classification of some residential regions as hybrid regions, which caused the lowest accuracy for residential regions. The most common misjudgments are classifying residential regions as hybrid regions, classifying hybrid regions as residential regions, and classifying work regions as hybrid regions. We could find that there are still some errors in identifying the boundaries of hybrid regions, and this is a good direction for future work.

Table 5 shows the confusion matrix of multi daily activities-based classification. Mostly the identification accuracy was higher than 0.8, only the greenbelt identification accuracy was 0.667; 33.3% greenbelts are classified as leisure entertainment, considering that some scenic spots have both entertainment and green land, in fact, they are acceptable to be classified into any category.

#### 4.4.4. Comparation with Other Researches

We compared similar research from the past five years, including city, data, and results, as shown in Table 6. It can be concluded that methods containing travel data (bicycle sharing and smart card) perform better. Using multi-sources data, our proposed method could adapt to two classification scenarios (jobs–housing-based classification and multi daily activities-based classification), other methods have not been proved this. The trend of functional region classification is to apply more dimensional data to solve more classification problems.

AUC denotes areas under the curve, and the types of each research are as follows.

Liu et al.: commercial sites, office building/space, transport facilities, others, government, education, residence communities, green space.

Wang et al.: Farmland, forest, grass, water, urban construction land, rural construction, other construction land, unused areas.

Zhai et al.: developed residential regions, developed work and industrial regions, developed commercial regions, emerging residential regions, emerging work and industrial regions, rural regions, nature parks and unknown regions.

Zhao et al.: residence, work, consumption, transit.

Proposed method: (1) residential regions, work regions, hybrid regions, transport regions. (2) science education (A), health (B), leisure entertainment (C), sport (D), greenbelt (E), residential regions (F), work regions (G) and transport regions (H).

## 5. Conclusions

Nowadays, big data analytics and deep learning methods are increasingly used in transportation and land planning. Meanwhile, more and more travel companies publish open source data. These changes make it possible to conduct relevant analyses and quantify the urban functional regions.

This paper proposes a two-stage framework and uses SCD, POI data, and OTO data to identify the functional regions of subway stations. In the passenger flow stage, the SCD features are extracted and convertedin to a feature map. Then this map is fed into an identification model to capture passenger flow pattern and a ResNet model is used to build a prediction model. In built environment stage, the POI and OTO features are extracted, including static information about buildings and dynamic information about passenger preference. A SAE-DNN model is built for suspected anomaly recognition. Finally, we connect the outputs of two stages and use a SoftMax function to make the final identification of the functional region. We compare our methods with other classical methods, change hyperparameter, and try different datasets.

Experimental results show that the method, the hyperparameter, and the dataset used in this paper have the best MSE and $R^{2}$, which means that this method achieves the best performance, and this model can be used in multiple scenarios. The classification results can help to better plan the surrounding areas of the subway stations, build supporting facilities to facilitate residents’ travel life, and construct smart cities. In the future, people can use this model to further optimize data collection, then to select areas for new subway stations and to plan for the upcoming subway stations, that is, to construct surrounding facilities in advance according to their corresponding functional area divisions.

In order to achieve better effects, there are some points we can improve. First, we need to solve the errors that appear when identifying the boundary of hybrid regions. Additionally, several different classification types need to be done to test this model. Next, in order to obtain more stable conclusions, we need to extend our analysis by training models based on data from different seasons. Finally, combining various kinds of travel data can be effective to weaken the impacts of random factors on the recognition results. Even though this study fails to collect other traffic data from the same periods (such as bus and bicycle sharing data), multivariate data fusion can greatly enhance the accuracy and reliability of this model, which has good application prospects.

## Figures and Tables

**Table 1:** Overall performance comparison. SAE–DNN: deep neural network with stacked autoencoders; LR: liner regression; RFR: random forest regression; SVR: support vector regression; DNNs: deep neural networks; CNNs: convolutional neural networks.

Method. (Stage1 + Stage2)	MSE	$R^{2}$	Method (Stage1 + Stage2)	MSE	$R^{2}$
LR + CNNs	77.59	0.59	SVR + ConvGRU	56.14	0.72
LR + ConvLSTM	72.39	0.61	SVR + ResNet	49.58	0.77
LR + ConvGRU	70.65	0.63	DNNs + CNNs	59.54	0.72
LR + ResNet	65.23	0.65	DNNs + ConvLSTM	54.28	0.78
RFR + CNNs	59.75	0.62	DNNs + ConvGRU	54.69	0.79
RFR + ConvLSTM	55.96	0.66	DNNs + ResNet	45.98	0.81
RFR + ConvGRU	54.29	0.68	SAE-DNNs + CNNs	50.87	0.79
RFR + ResNet	47.68	0.72	SAE-DNNs + ConvLSTM	48.85	0.83
SVR + CNNs	65.49	0.68	SAE-DNNs + ConvGRU	49.58	0.83
SVR + ConvLSTM	55.97	0.7	SAE-DNNs + ResNet	**40.25**	**0.86**

The bold denotes to the best performance.

**Table 2:** Comparison of the structure of SAE-DNNs.

Hidden Layers	Hidden Units	MSE	$R^{2}$
3	[100, 100, 100]	66.14	0.66
3	[200, 200, 200]	60.25	0.69
3	[400, 400, 400]	59.54	0.72
4	[100, 100, 100, 100]	46.28	0.78
4	[100, 200, 200, 100]	43.85	0.81
4	[200, 200, 200, 200]	42.36	0.83
4	[200, 400, 400, 200]	**40.25**	**0.86**
5	[400, 400, 400, 400]	41.89	0.85
5	[400, 800, 800, 400]	42.69	0.83

The bold denotes to the best performance.

**Table 3:** Performance comparisons of the 7 train datasets.

Dataset	SCD	POI	OTO	SCD + POI	SCD + OTO	POI + OTO	SCD + POI + OTO
MSE	52.08	54.94	65.32	46.98	49.58	48.34	**40.25**
$R^{2}$	0.76	0.74	0.69	0.81	0.78	0.80	0.86

The bold denotes to the best performance.

**Table 4:** Confusion matrix of jobs–housing-based classification.

Types	Residential Regions	Work Regions	Hybrid Regions	Transport Regions	Total	Accuracy
Residential regions *	65	2	11	0	78	0.833
Work regions *	3	92	10	0	105	0.876
Hybrid regions *	8	5	148	3	164	0.902
Transport regions *	0	0	2	9	11	0.818

* Denotes to ground truth.

**Table 5:** Confusion matrix of multi daily activities-based classification. Science education (A), health (B), leisure entertainment (C), sport (D), greenbelt (E), residential regions (F), work regions (G) and transport regions (H).

Types	A	B	C	D	E	F	G	H	Total	Accuracy
A *	43	3	0	0	0	0	4	0	50	0.86
B *	4	17	0	0	0	0	0	0	21	0.809
C *	0	0	68	5	6	0	0	0	79	0.861
D *	0	0	0	5	0	0	0	0	5	1
E *	0	0	4	0	8	0	0	0	12	0.667
F *	0	0	4	0	0	86	4	0	94	0.915
G *	3	0	3	0	0	2	78	0	86	0.907
H *	0	0	0	0	2	0	0	9	11	0.818

* Denotes to ground truth.

**Table 6:** Comparation with Other Researches.

Research	City	Data	Results
Liu et al. [41]	Chinese cities	OpenStreetMap, POI	Match degree = 58.1%
Wang et al. [42]	Zhanggong	GIS data	ACU = 61.6%
Zhai et al. [49]	Wuxi	POI, Truck, Mobile phone	Overall accuracy = 0.7424 ± 0.0016
Zhao et al. [44]	San Francisco	Bicycle sharing	$R^{2}\approx 0.9$ for each type
Proposed method	Beijing	Smart card, POI, OTO	$R^{2}=$ 0.86 and 0.89
